# Promoting resilience in adolescents: A new social identity benefits those who need it most

**DOI:** 10.1371/journal.pone.0210521

**Published:** 2019-01-10

**Authors:** Elizabeth Koni, Saleh Moradi, Hitaua Arahanga-Doyle, Tia Neha, Jillian G. Hayhurst, Mike Boyes, Tegan Cruwys, John A. Hunter, Damian Scarf

**Affiliations:** 1 Department of Psychology, University of Otago, Dunedin, New Zealand; 2 School of Psychology, Victoria University of Wellington, Wellington, New Zealand; 3 School of Physical Education, University of Otago, Dunedin, New Zealand; 4 Research School of Psychology, Australian National University, Australia; Harvard University, UNITED STATES

## Abstract

The Social Identity Approach to Health holds that groups provide us with a sense of meaning and belonging, and that these identity processes have a significant positive impact on our health and wellbeing. Typically, research drawing from the social identity approach with adolescents has focused on the benefits of existing group memberships. Here, using a sail-training intervention, we investigated the impact of providing adolescents with a new group (i.e., a new social identity) on psychological resilience. Across two studies, we demonstrate the benefits of a new social identity, in terms of increases in psychological resilience, flow predominantly to those adolescents who report the lowest levels of resilience at the start of the voyage. We discuss our findings in relation to the social identity approach and adolescent identity development more generally.

## Introduction

One of the primary tasks of adolescence is to find an answer to the question: “Who am I?” On the surface, this question might seem relatively straightforward. The processes that underlie identity development are, however, extremely complex. Crocetti, Rubini, and Meeus’ [1] three-factor model of personal identity development provides a relatively simple way of capturing these processes. The first factor, commitment, refers to the choices individuals have made in important life domains (e.g., education). The second factor, in-depth exploration, captures the degree to which individuals reflect on the various commitments they have enacted. The third factor, re-consideration of commitment, involves comparing current commitments with potential alternatives. Broadly, when combined, these three factors allow us to think of an adolescent at any given time as being in one of two identity cycles: identity formation (a combination of commitment and reconsideration) or identity maintenance (a combination of commitment and in-depth exploration) [2, 3]. Although capturing personal identity development, and the cycles therein, what this model neglects is the central role that *social identities* play in identity development [4]. Indeed, identity development is not simply an inside-out process; the outside also gets in.

A brief review of the adolescent identity literature makes clear that the field has focused on the development of personal identity almost to the exclusion of social identity [4–6]. This state of play is surprising, given that (1) the adolescent literature clearly demonstrates that peer (i.e., social) groups have a significant impact on adolescent development [7–11] and, (2) social identity theory makes clear that people tend to define their identity in terms of the groups they are part of, making personal and social identity intricately intertwined [4, 12, 13]. Beyond its conceptual utility in the identity domain, recent extensions of the social identity perspective may have important implications for the health and well-being of adolescents.

### The Social Identity Approach to Health

Derived from social identity theory [12] and self-categorisation theory [13], the Social Identity Approach to Health holds that groups provide us with a sense of meaning and belonging, and that these identity processes have a significant positive impact on our health and wellbeing [14–17]. Evidence in support of the social identity approach to health is rapidly building. For example, group memberships are protective against developing depression, can be curative of existing depression, and help to prevent depression relapse [14, 18–20]. With respect to adolescents, multiple group memberships serve as a protective factor during significant life transitions [21, 22], are an antecedent of self-esteem [23, 24], and are predictive of better mental health [25, 26].

Typically, research drawing from the social identity approach with adolescents has focused on the benefits of existing group memberships (e.g., family, school, etc.) [23, 25–28]. A smaller number of studies, however, have investigated the benefits of providing adolescents with the opportunity to join a new group and, thus, form a new social identity [29–32]. The current project builds upon this evidence by investigating the benefits of two sail training interventions in which adolescents undertake a 10-day (Study 1) or 7-day (Study 2) voyage. Several aspects of these voyages encourage the development of a new social identity. First, adolescents do not know one another prior to participating in the voyages, eliminating the possibility that cliques already exist. Second, smart phones are not allowed on board, limiting their ability to connect with existing social groups. Previously, we have demonstrated that this approach results in adolescents forming a strong group identity that this new social identity predicts increases in psychological resilience that persist for many months after the voyage [30, 33]. Although a slightly amorphous concept [34], in this study resilience was measured using a modified version of Wagnild and Young’s [35] resilience scale. The scale measures the ability to cope with life’s challenges and includes items that tap protective factors (e.g., “My life has meaning” and “I am friends with myself”) and items that tap persistence (e.g., “My belief in myself gets me through hard times”).

In the current study, we take our earlier work one-step further, and investigate whether these benefits flow predominantly to those adolescents who are more or less vulnerable at the beginning of the journey. Specifically, we posited two competing hypotheses, (Hypothesis 1) that benefits of the journey would flow primarily to those adolescents who start the voyage with high levels of resilience, or (Hypothesis 2) that benefits of the journey would be most pronounced for those adolescents most in need of a resilience boost (i.e., those who start the voyage with the lowest levels of resilience). Across both studies, we followed current recommendations in the field with regard to including control variables [36, 37] and considered controlling for the main effects of age, gender, and socioeconomic status. Preliminary analyses revealed that, regardless of their presence or absence, we found identical results. Thus, to maximize statistical power and offer the most interpretable results, we report the findings without control variables.

## Study 1

### Materials and methods

#### Participants

One hundred thirty-six adolescents (82 females) participated in the current study (*M* age = 16.58 years; *SD* = 1.73 years). All participants had taken part in a 10-day voyage on the Spirit of New Zealand. The Spirit of New Zealand is a barquentine that sails the coastal waters of New Zealand. The Spirit draws adolescents from around New Zealand and, although ethnicity data is not available for participants in the current study, operational data suggests that the demographic makeup of the voyages is typically representative of the New Zealand population (New Zealand European 74%, Māori (the indigenous people of New Zealand) 12%, Pacific Peoples 7%, Asian 3%, other 5%). Each voyage includes 30–40 adolescents. Participants are assigned to a Watch Group on the first day of the voyage and remain in this group throughout. The Watch Group consists of 5 males and 5 females and is the core social group formed during the voyage. Each day, the Watch Groups move to a different part of the vessel and complete a variety of goal-oriented activities (e.g., hoisting the sails) that can only be successfully achieved if they work together. At the end of each day, Watch Groups discuss what they gained from the day and how well they worked (or didn’t work) together. For a full description of the voyage, see Scarf, Moradi (30). The current study was reviewed and approved by the University of Otago Human Ethics Committee. Consent was obtained in written form.

#### Measures

Resilience was assessed at the start (Time 1) and end (Time 2) of the voyage using a shortened version of Wagnild and Young’s [35] Resilience Scale. This 15-item scale was adapted by Neill and Dias [38] to measure changes in resilience brought about by outdoor interventions (e.g., “My belief in myself gets me through hard times,” with response options from 1 = disagree strongly to 7 = agree strongly, *Cronbach’s alpha* = .87). Social identity was measured at Time 2 using Sheldon and Bettencourt’s [39] 3-item inclusion scale (e.g., “I feel a sense of belonging with my watch”) and Ellemers et al.’s [40] 3-item self-categorization sub-scale (e.g., “I identify with the other members of my watch,” with response options from 1 = disagree strongly to 5 = agree strongly, *Cronbach’s alpha* = .78).

### Results

**Fig 1:**
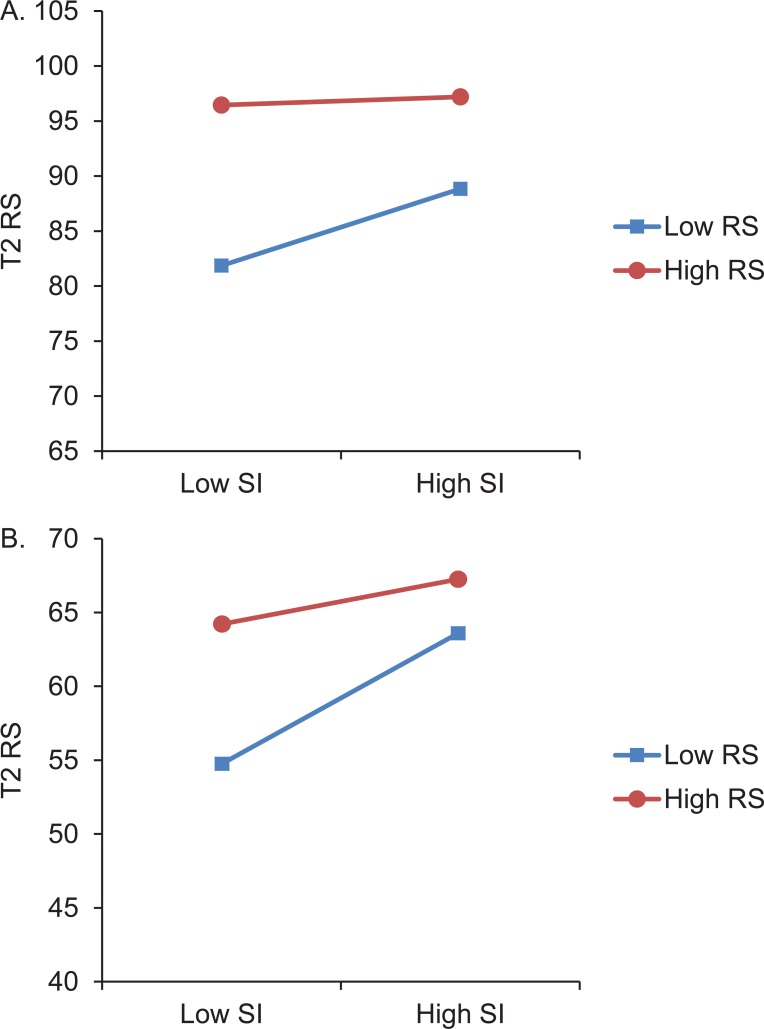
Youth lower in resilience at Time 1 display the greatest benefit of a new social identity. (A) Interaction between social identity (SI) and resilience (RS) at Time 1 (T1) on resilience at Time 2 (T2) for youth in Study 1. (B) Interaction between SI and RS at T1 on RS at T2 for youth in Study 2.

Table 1 presents the means, standard deviations, and correlations between all measures. OLS regression analyses revealed that both resilience prior to the voyage (i.e., Resilience at Time 1) and social identity showed significant positive main effects on resilience immediately after the voyage (i.e., Resilience at Time 2). Moreover, we found a significant interaction effect of Resilience at Time 1 and social identity on Resilience at Time 2 (see Table 2). The relationship between social identity and Resilience at Time 2 was stronger for participants low in Resilience at T1 than for participants high in Resilience at T1. Confirming our second hypothesis, a simple slope analysis showed a significant positive association between social identity and Resilience at T2 for participants low in Resilience at T1 (simple slope *B* = 3.47, *t*(132) = 5.52, *p* < .001) while no such relationship existed for participants high in Resilience at T1 (simple slope *B* = .36, *t*(132) = .36, *p* = .72). The interaction is shown in Fig 1.

**Table 1:** Means, standard deviations, and correlations for all variables in Study 1.

	*M*	*SD*	**1**	**2**	**3**
1. Resilience at T1	83.34	9.64	-		
2. Social Identity	37.38	4.61	.46**	-	
3. Resilience at T2	90.44	10.90	.72**	.59**	-

*Notes*. *N* = 136. Cronbach's Alphas are represented between brackets on the main diagonal.

** *p* < .01.

**Table 2:** Results for analyses regressing Resilience at T1 and Social Identity on Resilience at T2.

	Model 1			Model 2		
	*B*	*SE*	*β*	*B*	*SE*	*β*
Resilience at T1	6.25	.67	.57**	5.74	.64	.53**
Social Identity	3.60	.67	.33**	1.93	.75	.18*
Resilience at T1 × Social Identity				-1.56	.38	-.28**
Overall *R*^*2*^	.61			.65		
Overall *F*	103.57**			83.27**		
*Df*	133			132		

*Notes*. *N* = 136. The table presents the unstandardized b-coefficients and standard errors for centered variables.

* *p* < .05.

** *p* < .01.

### Discussion

The finding that the relationship between social identity and Resilience at T2 was stronger for participants low in Resilience at T1 than for participants high in Resilience at T1 provides initial evidence that the acquisition of a new social identity benefits those who need it the most. In Study 2, utilizing data drawn from Arahanga-Doyle, Moradi (32), we extend the analyses in Study 1 by applying them to an independent sample of adolescents that have taken part in a 7-day voyage on the R. Tucker Thompson. The R. Tucker Thompson is a traditional gaff-rigged schooner based in the Bay of Islands, Northland, New Zealand. In contrast to the Spirit, the Tucker draws youth exclusively from the Northland region of New Zealand, an area characterised by a number of low socio-economic indicators (e.g., low educational achievement, high unemployment, etc.). Thus, the Tucker provides an important test regarding whether the effects observed in Study 1 hold for adolescents from more challenging backgrounds.

## Study 2

### Method

#### Participants

Participants in the current study were 91 adolescents (55 females); *M* age = 15.25 years; *SD* = 1.18 years). Of those, 54 self-identified as Māori and 37 as New Zealand European. All participants took part in a 7-day youth voyage on the R. Tucker Thompson. Ten to 12 adolescents participate in each voyage. For a full account of the sample and the voyage see Arahanga-Doyle, Moradi (32). Given that the majority of adolescents who take part in the voyage identify as Māori, Māori concepts and practises play a central role in the voyage. For example, before adolescents step on board the boat there is first a *mihi* (welcome) and then *whanaungatanga* (belonging) is established by way of a young crewmember facilitating a set of games and activities that provide the opportunity for the youth to talk to one another and become comfortable with one another. As Macfarlane and colleagues [41] note *whanaungatanga* is “…both a sense of belonging to and a sense of relating to others, within a context of collective identity and responsibility” (p. 107). These pre-voyage games and activities are run until awkwardness is seen to disappear and ensure that when adolescents step on board the Tucker they do so as a collective or group. The current study was reviewed and approved by the University of Otago Human Ethics Committee. Consent was obtained in written form.

#### Measures

Resilience was assessed using 10-items drawn from Wagnild and Young’s [35] Resilience Scale. The 10-items were based on those used by Neil and Dias’ [38] to measure changes in resilience brought about by outdoor interventions (e.g., ‘I usually manage one way or another’, Cronbrach’s α = .95). Social identity was measured using a single-item social identification measure [42] (i.e., ‘I identify with other members of my voyage group’) combined with all three items from Sheldon and Bettencourt’s [39] inclusion scale (e.g., ‘I feel a sense of belonging with this voyage group’, Cronbrach’s α = .91).

### Results

Table 3 presents the means, standard deviations, and correlations between all measures. Similar to Study 1, OLS regression analyses revealed significant positive main effects for both resilience prior to the voyage (i.e., Resilience at Time 1) and social identity on resilience immediately after the voyage (i.e., Resilience at Time 2). As hypothesized, the interaction effect of Resilience at Time 1 and social identity on Resilience at Time 2 was also significant (see Table 4). Again confirming our second hypothesis, the relationship between social identity and Resilience at Time 2 was stronger for participants low in Resilience at Time 1 than for participants high in Resilience at Time 1. This confirmed H2. Nevertheless, an additional simple slope analysis showed significant positive association between social identity and Resilience at Time 2 for participants both low (simple slope *B* = 4.73, *t*(87) = 6.90, *p* < .001), and high (simple slope *B* = 2.32, *t*(87) = 3.46, *p* < .001) in Resilience at Time 1. The interaction is shown in Fig 1.

**Table 3:** Means, standard deviations, and correlations for all variables in Study 2.

	*M*	*SD*	**1**	**2**	**3**
1. Resilience at T1	55.60	8.61	-		
2. Social identity	25.69	3.91	.12	-	
3. Resilience at T2	62.22	7.23	.55**	.52**	-

*Notes*. *N* = 91. Cronbach's Alphas are represented between brackets on the main diagonal.

** *p* < .01.

**Table 4:** Results for analyses regressing Resilience at T1 and Social Identity on Resilience at T2.

	Model 1			Model 2		
	*B*	*SE*	*β*	*B*	*SE*	*β*
Resilience at T1	3.49	.56	.48**	3.28	.54	.45**
Social Identity	3.12	.56	.43**	2.97	.54	.41**
Resilience at T1 × Social Identity				-1.45	.50	-.22**
Overall *R*^*2*^	.49			.53		
Overall *F*	41.95**			33.15**		
*Df*	88			87		

*Notes*. *N* = 91. The table presents the unstandardized b-coefficients and standard errors for centered variables.

** *p* < .01.

### Discussion

The findings of Study 2 largely replicated those of Study 1. The relationship between social identity and Resilience at T2 was stronger for participants low in Resilience at T1 than for participants high in Resilience at T1. In contrast to Study 1, the relationship between social identity and Resilience at T2 held for participants high in Resilience at T1. This latter finding may reflect the fact that, in this more vulnerable population, even adolescents considered high in resilience still have room for growth.

## General discussion

In two studies, the benefits of social identity for resilience at the end of a developmental voyage was stronger for adolescents low in resilience at the start of the voyage, relative to adolescents high in resilience at the start of the voyage. With respect to the basic relationship between social identity and resilience, these findings are consistent with previous work investigating the social identity approach and a range of positive outcomes in adolescents [21–23, 25–28]. This study, however, extends this earlier work, first by demonstrating that the benefits of a new social identity hold for both normative adolescent groups (Study 1) and those facing significant challenges (Study 2), and second by demonstrating that the benefits of a new social identity may be the most marked for adolescents within these groups that come to the voyage with the lowest levels of resilience.

The findings of Study 2 deserve particular attention given the majority of adolescents that take part in these voyages on the Tucker are Māori, the indigenous people of New Zealand. A large body of work demonstrates that the mental health challenges faced by adolescents in New Zealand disproportionately fall on Māori. For example, relative to non-Māori adolescents, Māori adolescents experience significantly higher rates of depression and anxiety [43]. The profound benefits of a new social identity for Māori youth likely reflects the fact that the central concepts of the social identity approach are also central concepts in *Te Ao Māori* (a Māori worldview). More specifically, they are captured by the terms whānau and whanaungatanga, which “…indicate both a sense of belonging to and a sense of relating to others, within a context of collective identity and responsibility” [41]. The current study highlights the fact that these concepts should be central to any approaches or interventions that aim to improve the mental health of Māori youth.

It is important to address two alternative explanations for the findings of the current study. First, given resilience was measured on only the first and last day of the voyage, it is possible that the improvement displayed by youth low in resilience at Time 1 merely reflects regression toward the mean. That is, given it was the first day of the voyage and some youth may have been nervous about the voyage, the Time 1 scores may be an extreme value. Although we cannot rule this possibility out, we have demonstrated in our previous work that resilience scores on the first day of the voyage are comparable to scores measured in the same individuals up to a month before the voyage [30], suggesting there is little reason to believe the Time 1 scores in the current study are extreme. A second alternative explanation is that, rather than youth low in resilience at Time 1 benefiting the most from a new social identity, the relatively stable level of resilience displayed by youth high in resilience at Time 1 reflects a ceiling effect. Again, the possibility is unlikely due to the fact the mean score for youth in Study 1 was still well below the maximum possible score and, with respect to Study 2, the youth high in resilience at Time 1 also displayed an increase in resilience over the course of the voyage.

### Limitations and future research directions

The current research is not without its limitations. First, although we have demonstrated the potential applicability of the social identity approach to adolescent health and wellbeing, it continues the tradition of investigating the personal and social components of identity in isolation [44]. To address this issue, future studies could assess the identity status of adolescents before and after they take part in the voyage. Indeed, assessing personal identity before the voyage would allow one to assess whether adolescents currently in the identity formation cycle (a combination of commitment and reconsideration) gain more from the voyage (i.e., the acquisition of a new social identity) relative to adolescents in the identity maintenance cycle (a combination of commitment and in-depth exploration) [2, 3]. Similarly, assessing personal identity after the voyage would reveal whether the acquisition of a new identity has shifted adolescents’ identity status. A second limitation of the current study is the absence of a long-term follow-up assessment. Previously, we demonstrated that the increases observed following the Spirit voyage are maintained 9 months after the voyage [30], which suggested that the benefits found for the current sample are unlikely to be short-lived. With respect to the Tucker voyage, the adolescents that take part require considerable support to complete the Time 1 and Time 2 questionnaires, making our standard approach to a long-term follow-up assessment (i.e., mailing adolescents questionnaires to complete at home) inappropriate. One potential solution to this issue would be to conduct follow-up assessments via phone.

### Conclusion

Across two studies, our findings clearly demonstrate that a new social identity *does* benefit those adolescents who need it most. Although sail training voyages represent one-shot experiences, and thus may be viewed as less likely to lead to long-term change, it is important to remember that sail training leads to the formation of especially meaningful and long-lasting bonds among adolescents. These bonds, built on the foundation of shared sailing experiences, albeit for a brief period of time, can indeed have a profound effect on adolescents’ sense of self and social identity.

## Supporting information

S1 Data Dataset for Study 1. (SAV)

S2 Data Dataset for Study 2. (SAV)
